# Supplementary material for: Mechanism of action and therapeutic route for a muscular dystrophy caused by a genetic defect in lipid metabolism
Source: Nat Commun. 2022 Mar 23;13:1559. doi: 10.1038/s41467-022-29270-z (PMC8943011; doi:10.1038/s41467-022-29270-z)
Supplement: Supplementary file 1 — Supplementary Information [file 41467_2022_29270_MOESM1_ESM.pdf]

Supplementary Information

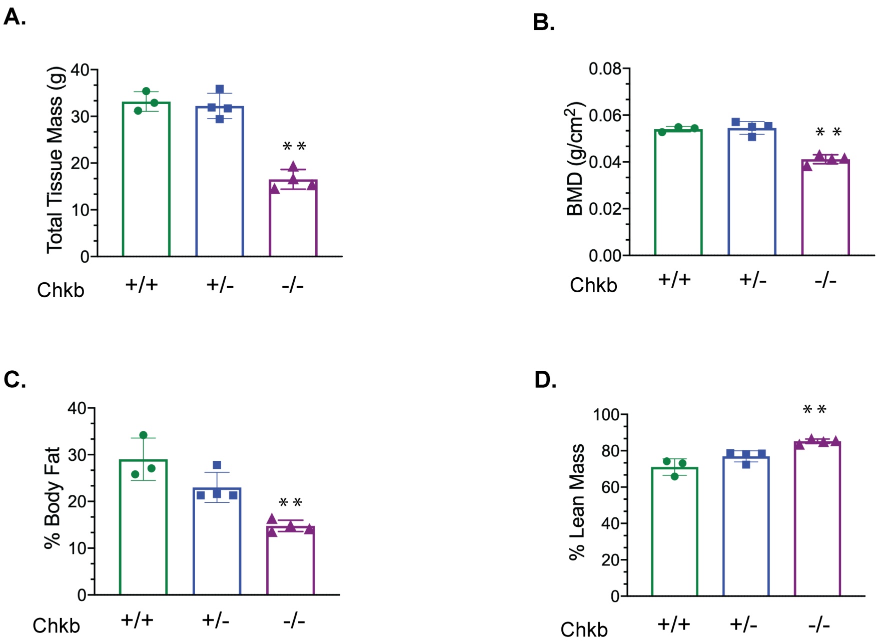

Supplementary Figure 1. Body composition analysis in choline kinase deficient mice. Total tissue mass **(A),** bone mineral density (BMD) **(B)**, percent body fat **(C)** and percent lean mass **(D)** were measured using DEXA analysis. Values expressed as means ±SEM.
For (**A**-**D**), n = 3 independent (*Chkb*$^{+/+}$), n = 4 independent (*Chkb*$^{+/-}$) and n = 4 inependent (*Chkb*$^{-/-}$) mice. One-way ANOVA with Tukey's multiple comparison test; $p < 0.0001$. **P <0.001 comparing wild-type with knockout mice and heterozygous with knockout mice.

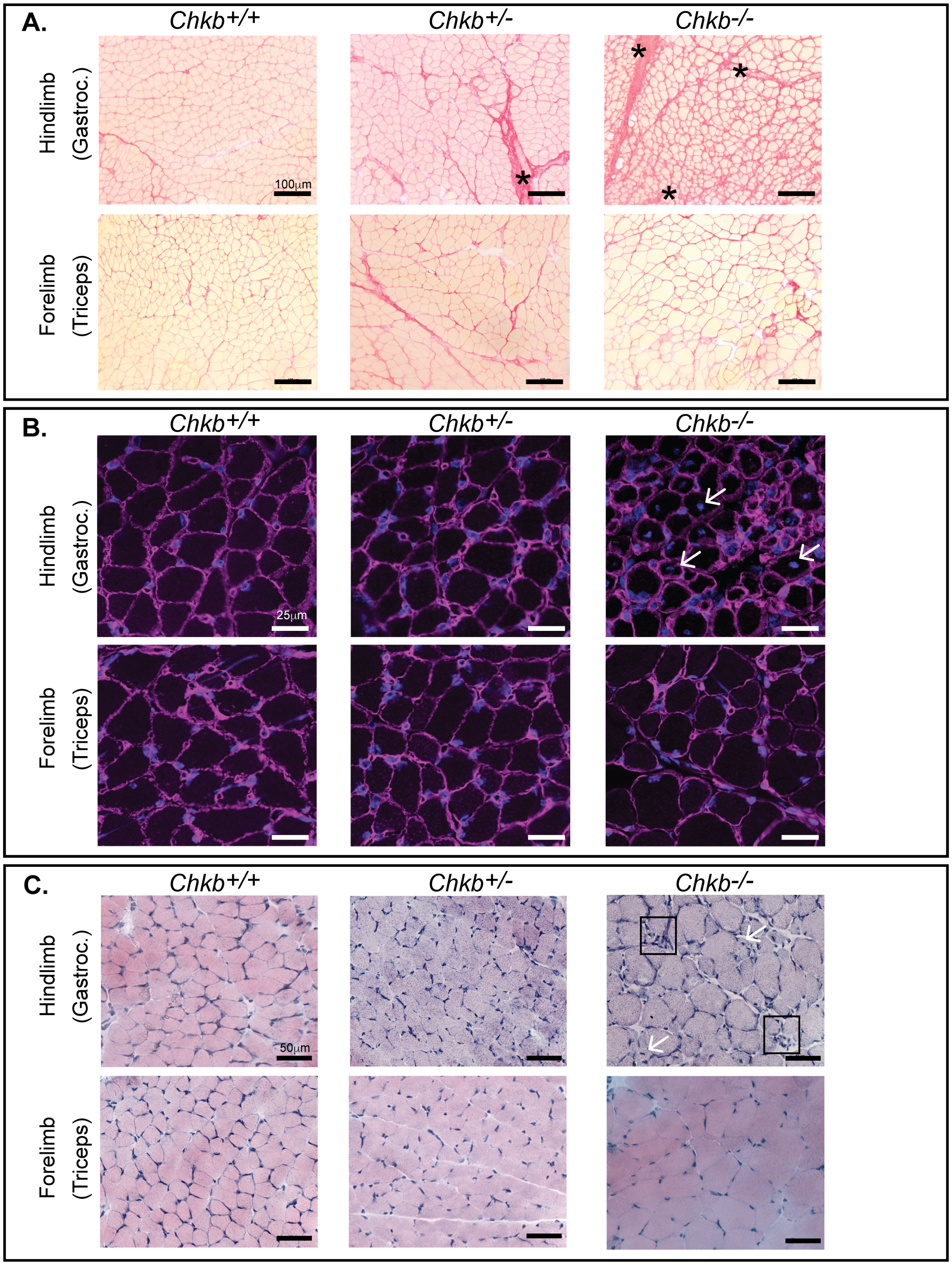

Supplementary Figure 2. Early signs of muscular dystrophy in hindlimb skeletal muscle from *Chkb*$^{-/-}$ mice. **(A)** Representative picrosirius red-stained cross sections of forelimb (Triceps) and hindlimb (Gastrocnemius) of 3 independent *Chkb*$^{+/+}$, *Chkb*$^{+/-}$ and *Chkb*$^{-/-}$ mice at 25 days of age. Scale bar = 100 µm (**B**) Representative confocal immunofluorescence microscopy for laminin in cross sections of forelimb (Triceps) and hindlimb (Gastrocnemius) of 3 independent *Chkb*$^{+/+}$, *Chkb*$^{+/-}$ and *Chkb*$^{-/-}$ mice at 25 days of age showing the large variation among fiber sizes, a rounded shape of atrophic fibers and increased central nucleation in dystrophic hindlimb muscles from *Chkb*$^{-/-}$ mice. Dapi was used to stain nuclei blue. Scale bar = 25 µm (**C**) Representative H&E staining of cross sections of forelimb (Triceps) and hindlimb (Gastrocnemius) of 3 independent *Chkb*$^{+/+}$, *Chkb*$^{+/-}$ and *Chkb*$^{-/-}$ mice at 25 days of age. Scale bar = 50 µm. *=red-stained fibrillary elements. White arrow=Central nucleation. Black square shows infiltration of mononuclear inflammatory cells.

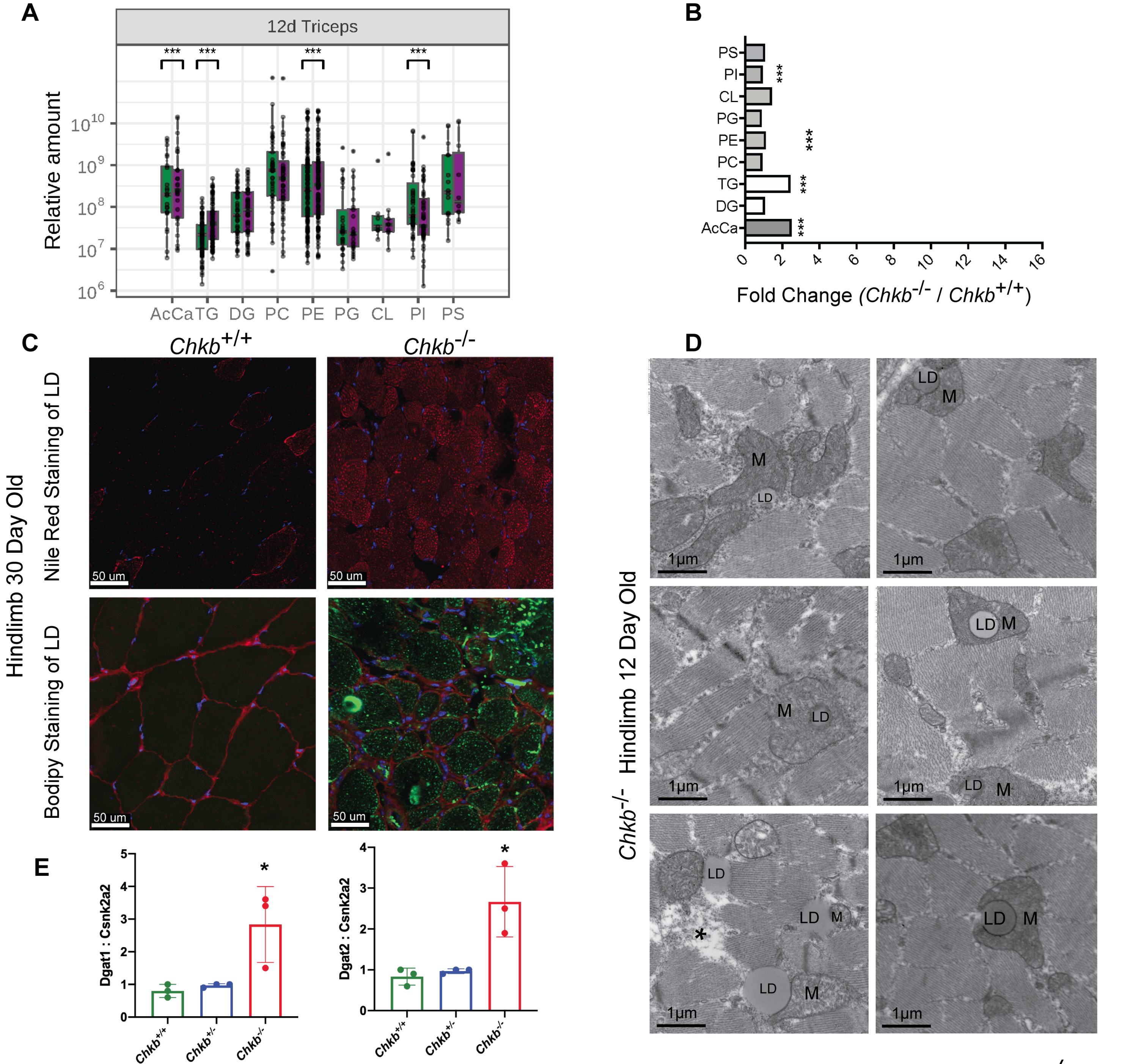

Supplementary Figure 3. Increased intramyocellular lipid droplet accumulation in skeletal muscles from *Chkb*$^{-/-}$ mice. **(A)** Comparison of expression levels of major glycerophospholipids and AcCa between the *Chkb*$^{+/+}$ and *Chkb*$^{-/-}$ mice; 12 days old forelimb (triceps). Boxplots bounds (hinges) correspond to the 25th and 75th percentiles of the data with the boxplot center corresponding to the median value. The upper/lower whiskers extend from the hinges to the largest/smallest value no further than 1.5 times the interquartile range away from the hinges. n=3 independent Chkb+/+ and 3 independent Chkb-/- mice.

(**B**) Summary of fold change and statistical tests performed on major glycerophospholipids For (A-D), n=3 independent Chkb+/+ and 3 independent Chkb-/- mice. Pairwise Wilcoxon signed rank test with Bonferroni correction was used to determine the significance of a median pair-wise fold-increase in lipid amounts at an overall significance level of 5%. All statistical tests were two-sided. As the Bonferroni correction is fairly conservative, significant differences are reported at both pre-correction (*) and post-correction (***) significance levels. p = 0.000015 (AcCa), p = 0.0.38507 (DG), p = 0.0000 (TG), p = 0.10484 (PC), p = 0.000938 (PE), p = 0.088545 (PG), p = 0.0000 (PI), p = 0.128906 (PS).

(**C**) Quadriceps muscle sections of 30 days old *Chkb*$^{+/+}$ and *Chkb*$^{-/-}$ mice were fixed and stained with Nile Red-550 / 640 nm to visualize LDs (Red) (Upper Pannel) or with BODIPY-493/503 to visualize LDs (Green) and Concanavalin A dye conjugate (CF™ 633) to stain membrane (Red). Dapi was used to stain nucleus (Blue). Representative of 3 mice with similar results. For (**C**) Scale bar = 50 μm. (**D**) Transmission electron microscopy (TEM) appearance of the hindlimb muscle samples of 12 days old *Chkb*$^{-/-}$ mice showing the high prevalence of peri-droplet mitochondria in hindlimb muscles. Representative of 3 mice per group. LD=Lipid droplets. M=Mitochondria. *=Disrupted sarcomeres. For (**D**) Scale bar = 1 μm **(E)** Relative gene expression of the triglyceride synthesis enzymes DGAT1 and DGAT2.Values are means ± SD; n=3 mice per group. One-way ANOVA with Tukey's multiple comparison test. p = 0.0186 (Dgat1) and p = 0.0082 (Dgat2). *P< 0.05 vs all the other groups.

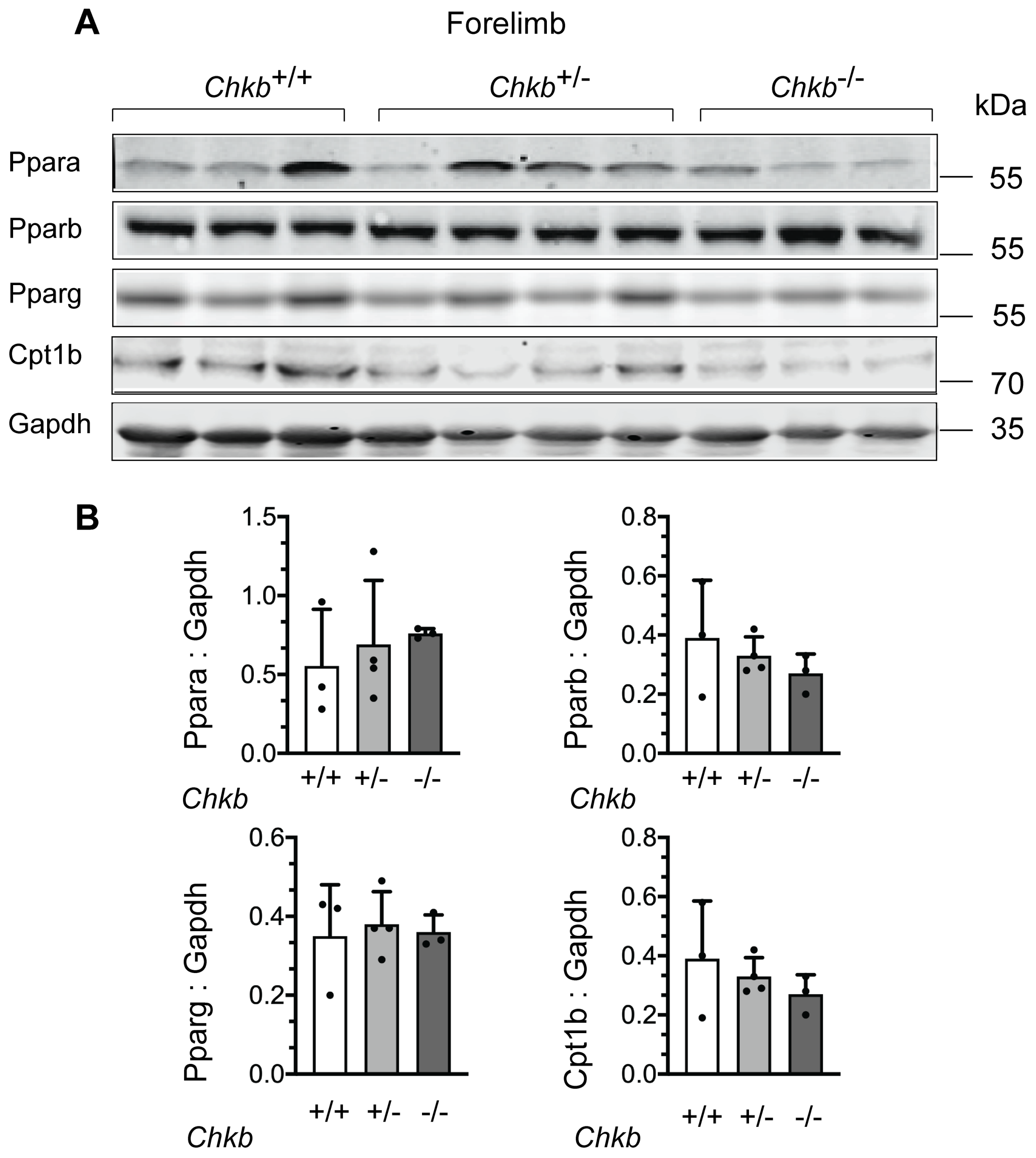

Supplementary Figure 4. Protein expression of the members of the Ppar family in forelimb samples. (**A**) Western blot of forelimb (Triceps) samples from three distinct (lanes 1–3) *Chkb*$^{+/+}$, four distinct (lanes 4–7) *Chkb*$^{+/-}$ and three distinct (lanes 8–10) *Chkb*$^{-/-}$ mice probed with anti-Ppara, anti- Pparb, anti-Pparg, anti- Cpt1b and anti-Gapdh antibodies. . For (**A**), n = 3 (*Chkb*$^{+/+}$), n = 4 (*Chkb*$^{+/-}$) and n = 3 (*Chkb*$^{-/-}$) mice per group. (**B**) densitometry of the WB data shows the ratio of Ppara, Pparb, Pparg and Cpt1b to Gapdh.Values are means ± SD; For (**B**), n=3 (*Chkb*$^{+/+}$), n = 4 (*Chkb*$^{+/-}$) and n = 3 (*Chkb*$^{-/-}$) mice per group. One-way ANOVA with Tukey's multiple comparison test, p = 0.7437 (Ppara), p = 0.4948 (Pparb), p = 0.9065 (Pparg). p = 0.4948 (Cpt1b). No significant difference was obserd among groups.

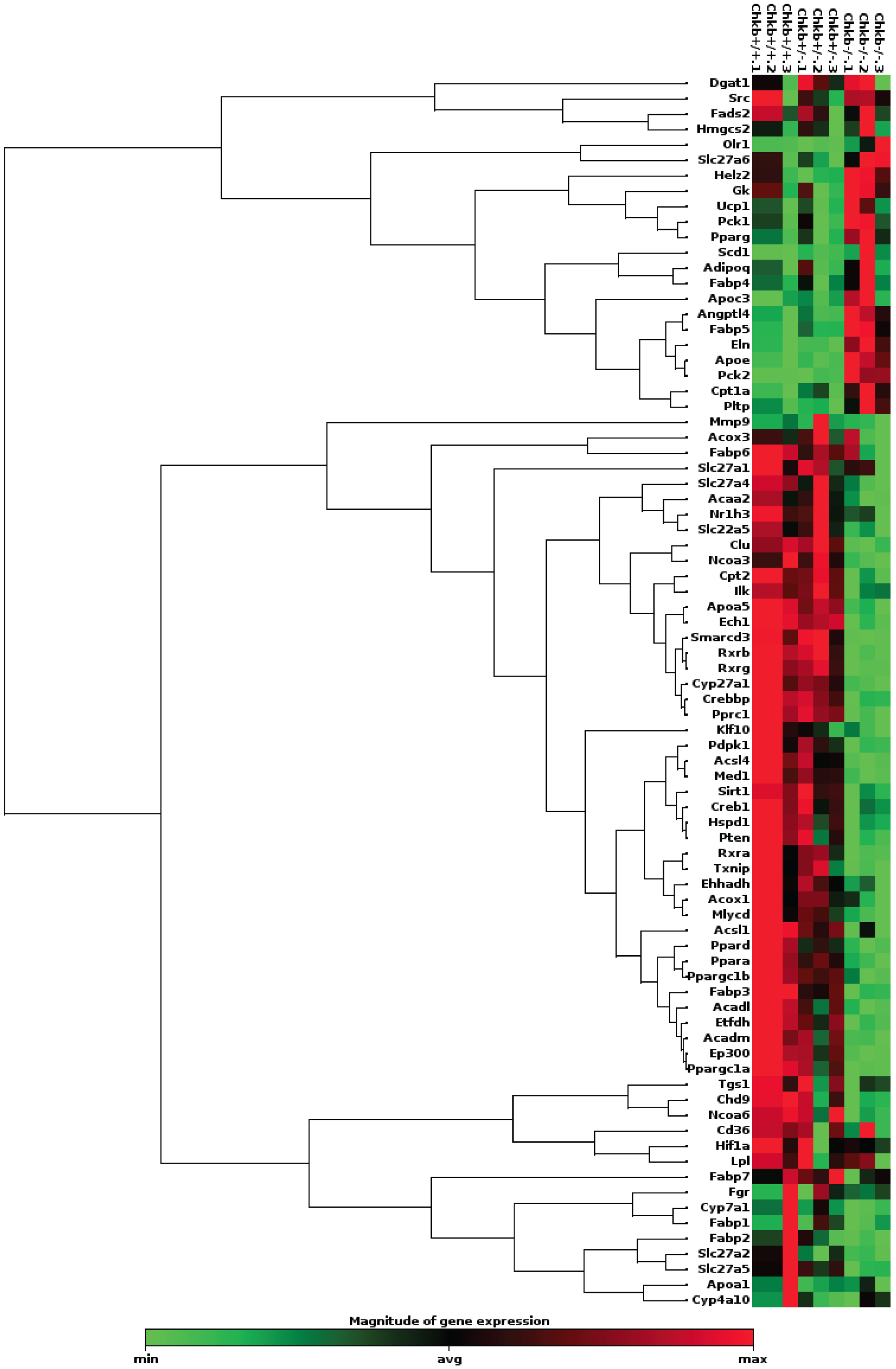

Supplementary Figure 5. Chkb regulates the expression of the members of the Ppar family as well as Ppar target genes. Clustergram showing non-supervised hierarchical clustering of the entire dataset to display a heat map with dendrograms indicating co-regulated genes across groups or individual samples. Sample Dimension: 1D. Join Type: Average. Color Coded: Average Genes.

Supplementary Table 1. Ppar associated genes under-expressed in $Chkb^{-/-}$ vs. $Chkb^{+/+}$ hindlimb

Gene Symbol	Fold Regulation	p-Value	Description
Rxrg	-16.38	0.000250	Retinoid X receptor gamma
Smarcd3	-14.40	0.000951	SWI/SNF related, matrix associated, actin dependent regulator of chromatin, subfamily d, member 3
Rxra	-8.78	0.008824	Retinoid X receptor alpha
Ppargc1a	-8.46	0.000002	Peroxisome proliferative activated receptor, gamma, coactivator 1 alpha
Cpt1b	-7.93	0.002692	Carnitine palmitoyltransferase 1b, muscle
Pparb	-6.19	0.000176	Peroxisome proliferator activator receptor delta
Cyp27a1	-5.58	0.001538	Cytochrome P450, family 27, subfamily a, polypeptide 1
Slc27a5	-5.43	0.022345	Solute carrier family 27 (fatty acid transporter), member 5
Hspd1	-5.05	0.001198	Heat shock protein 1 (chaperonin)
Acadm	-4.95	0.000659	Acyl-Coenzyme A dehydrogenase, medium chain
Rxrb	-4.80	0.000043	Retinoid X receptor beta
Txnip	-4.68	0.008384	Thioredoxin interacting protein
Crebbp	-4.54	0.000152	CREB binding protein
Ppara	-4.42	0.000517	Peroxisome proliferator activated receptor alpha
Etfdh	-4.39	0.000073	Electron transferring flavoprotein, dehydrogenase
Apoa5	-4.07	0.000064	Apolipoprotein A-V
Ep300	-3.89	0.000062	E1A binding protein p300
Fabp3	-3.75	0.000022	Fatty acid binding protein 3, muscle and heart
Acsl3	-3.33	0.000350	Acyl-CoA synthetase long-chain family member 3
Ppargc1b	-3.25	0.001233	Peroxisome proliferative activated receptor, gamma, coactivator 1 beta
Ech1	-3.24	0.000023	Enoyl coenzyme A hydratase 1, peroxisomal
Tgs1	-3.23	0.028174	Trimethylguanosine synthase homolog (S. cerevisiae)
Acsl4	-3.15	0.000615	Acyl-CoA synthetase long-chain family member 4
Med1	-3.10	0.001997	Mediator complex subunit 1
Pdpk1	-3.08	0.008230	3-phosphoinositide dependent protein kinase 1
Creb1	-3.02	0.003162	CAMP responsive element binding protein 1
Pprc1	-3.02	0.000136	Peroxisome proliferative activated receptor, gamma, coactivator-related 1
Slc27a2	-2.98	0.017603	Solute carrier family 27 (fatty acid transporter), member 2
Chd9	-2.89	0.000078	Chromodomain helicase DNA binding protein 9
Mlycd	-2.89	0.010148	Malonyl-CoA decarboxylase
Ncoa6	-2.86	0.000177	Nuclear receptor coactivator 6
Sirt1	-2.79	0.001001	Sirtuin 1 (silent mating type information regulation 2, homolog) 1
Cpt2	-2.77	0.002056	Carnitine palmitoyltransferase 2
Acsl1	-2.67	0.006296	Acyl-CoA synthetase long-chain family member 1
Acadl	-2.54	0.000127	Acyl-Coenzyme A dehydrogenase, long-chain
Clu	-2.52	0.000146	Clusterin
Acaa2	-2.48	0.010777	Acetyl-Coenzyme A acyltransferase 2 (mitochondrial 3-oxoacyl-Coenzyme A thiolase)
Klf10	-2.42	0.009957	Kruppel-like factor 10
Ehhadh	-2.34	0.022919	Enoyl-Coenzyme A, hydratase/3-hydroxyacyl Coenzyme A dehydrogenase
Pten	-2.30	0.000522	Phosphatase and tensin homolog
Slc22a5	-2.13	0.010148	Solute carrier family 22 (organic cation transporter), member 5
Slc27a4	-2.11	0.001068	Solute carrier family 27 (fatty acid transporter), member 4
Acox1	-2.04	0.034786	Acyl-Coenzyme A oxidase 1, palmitoyl
Nr1h3	-2.02	0.020303	Nuclear receptor subfamily 1, group H, member 3

Fold Regulation cut off =2. p-Value cut off= 0.05. Fold-Change (2^ (- Delta Delta CT)) is the normalized gene expression (2^(- Delta CT)) in the Test Sample divided the normalized gene expression (2^ (- Delta CT)) in the Control Sample. Fold-change values less than one indicate a negative or down-regulation, and the fold-regulation is the negative inverse of the fold-change. The p values are calculated based on a two-sided student's t-test of the replicate 2^ (- Delta CT) values for each gene in the control group and Chkb deficient groups.

Supplementary Table 2. Ppar associated genes over-expressed in $Chkb^{-/-}$ hindlimb vs. $Chkb^{+/+}$

Gene Symbol	Fold Regulation	p-Value	Description
Pck2	8.55	0.000216	Phosphoenolpyruvate carboxykinase 2 (mitochondrial)
Apoe	6.77	0.000851	Apolipoprotein E
Angptl4	4.46	0.007248	Angiopoietin-like 4
Eln	3.46	0.002955	Elastin
Fabp5	2.74	0.009434	Fatty acid binding protein 5, epidermal
Pltp	2.61	0.030470	Phospholipid transfer protein
Cpt1a	2.29	0.008722	Carnitine palmitoyltransferase 1a, liver
Pparg	2.24	0.041818	Peroxisome proliferator activated receptor gamma

hindlimb

Fold Regulation cut off =2. p-Value cut off= 0.05. Fold-Change (2^ (- Delta Delta CT)) is the normalized gene expression (2^(- Delta CT)) in the Test Sample divided the normalized gene expression (2^ (- Delta CT)) in the Control Sample. Fold-change values more than one indicate a positive or up-regulation. The p values are calculated based on a two-sided student's t-test of the replicate 2^ (- Delta CT) values for each gene in the control group and Chkb deficient groups.
