# Supplementary material for: Mechanism of action and therapeutic route for a muscular dystrophy caused by a genetic defect in lipid metabolism
Source: Nat Commun. 2022 Mar 23;13:1559. doi: 10.1038/s41467-022-29270-z (PMC8943011; doi:10.1038/s41467-022-29270-z)
Supplement: Supplementary file 3 — Reporting Summary [file 41467_2022_29270_MOESM3_ESM.pdf]

nature portfolio

Corresponding author(s): Christopher McMaster

Last updated by author(s): Jan 31, 2022

Reporting Summary

Nature Portfolio wishes to improve the reproducibility of the work that we publish. This form provides structure for consistency and transparency in reporting. For further information on Nature Portfolio policies, see our Editorial Policies and the Editorial Policy Checklist.

Statistics

For all statistical analyses, confirm that the following items are present in the figure legend, table legend, main text, or Methods section.

n/a	Confirmed	
☐	☒	The exact sample size (*n*) for each experimental group/condition, given as a discrete number and unit of measurement
☐	☒	A statement on whether measurements were taken from distinct samples or whether the same sample was measured repeatedly
☐	☒	The statistical test(s) used AND whether they are one- or two-sided<br>*Only common tests should be described solely by name; describe more complex techniques in the Methods section.*
☐	☒	A description of all covariates tested
☒	☐	A description of any assumptions or corrections, such as tests of normality and adjustment for multiple comparisons
☐	☒	A full description of the statistical parameters including central tendency (e.g. means) or other basic estimates (e.g. regression coefficient) AND variation (e.g. standard deviation) or associated estimates of uncertainty (e.g. confidence intervals)
☐	☒	For null hypothesis testing, the test statistic (e.g. *F*, *t*, *r*) with confidence intervals, effect sizes, degrees of freedom and *P* value noted<br>*Give P values as exact values whenever suitable.*
☒	☐	For Bayesian analysis, information on the choice of priors and Markov chain Monte Carlo settings
☒	☐	For hierarchical and complex designs, identification of the appropriate level for tests and full reporting of outcomes
☒	☐	Estimates of effect sizes (e.g. Cohen's *d*, Pearson's *r*), indicating how they were calculated

Our web collection on statistics for biologists contains articles on many of the points above.

Software and code

Policy information about availability of computer code

Data collection

Thermo Scientific™ LipidSearch™ software version 4.2 was used for lipid identification and quantification. An in-house script written in R (version 4.0.2) was used for data QC analysis, normalization and plotting.

Targeted metabolomics analysis was performed using high performance liquid chromatography coupled to a linear ion trap triple-quadrupole tandem mass spectrometer (LC-MS/MS) as previously described [https://doi.org/10.1111/tpj.14782]. Peak integration was performed using Skyline, an open-source multiple reaction monitoring (MRM) analysis software (version 21.0) [MacLean B, Tomazela DM, Shulman N, et al. Skyline: an open source document editor for creating and analyzing targeted proteomics experiments. Bioinformatics. 2010;26:966-968.]. An in-house script written in R (version 4.0.2) was used for data QC analysis, normalization and plotting. Briefly, for each metabolite, the peak intensities of samples were normalized to the mean of the corresponding intensities of the two nearest flanking QC pool samples (which consisted of a mix of all samples included in the analysis) to reduce drift.

For qPCR analysis, LightCycler® 96 Instrument Software, Version 1.1.1 was used.

Agilent Seahorse Wave Desktop software version 2.6 was used to transforms complex cellular metabolism data into publishable results.

Data analysis

All the graphs, calculations, and statistical analyses were performed using GraphPad Prism software version 8.0 for Mac (GraphPad Software, San Diego, CA, USA).
All image analysis were performed by Image J software, (Fiji) ImageJ 2.0.0-rc-69/1.52n, Build:269a0ad53f. Date : 2018-12-04T11:30:09+0000, Open source image processing software, Copyright 2010 - 2022.

For manuscripts utilizing custom algorithms or software that are central to the research but not yet described in published literature, software must be made available to editors and reviewers. We strongly encourage code deposition in a community repository (e.g. GitHub). See the Nature Portfolio guidelines for submitting code & software for further information.

Data

Policy information about availability of data

All manuscripts must include a data availability statement. This statement should provide the following information, where applicable:

- Accession codes, unique identifiers, or web links for publicly available datasets
- A description of any restrictions on data availability
- For clinical datasets or third party data, please ensure that the statement adheres to our policy

Data and/or Code Availability
All data that support the findings of this study and the custom code used during this study are available from the corresponding authors upon reasonable request.

Field-specific reporting

Please select the one below that is the best fit for your research. If you are not sure, read the appropriate sections before making your selection.

☒ Life sciences ☐ Behavioural & social sciences ☐ Ecological, evolutionary & environmental sciences

For a reference copy of the document with all sections, see nature.com/documents/nr-reporting-summary-flat.pdf

Life sciences study design

All studies must disclose on these points even when the disclosure is negative.

Sample size

Over the past 5-6 years we have extensively published on sample size calculations for complementary disease models (See references 1, 2 and 3 below). We have estimated the number of animals by performing power analysis based upon previous data using C57BL/10 and Mdx mice. Two different functional tests that are complementary to each other were chosen to systematically phenotype the transgenic mouse strains. Some of these tests are very sensitive (e.g., Fore limb grip strength require 3 mice to achieve a 20% effect size; See below) and some are modest (Behavioral activity measurements require 7 mice to achieve 20% effect size). Therefore, the number of animals needed to produce significantly meaningful data varies. Based on the power calculation, 6-7 mice/group are enough to get meaningful data in this study.

Parameter	C57BL/10 (N=14) Mean+/-SD	Mdx (N=10) Mean+/-SD	P-value	20% effect size	20% effect size*
GSM: Forelimb	0.0055 ± 0.0003	0.0044 ± 0.0004	<0.0001	3	
Hindlimb	0.0073 ± 0.0004	0.0063 ± 0.0005	<0.0001	7	
Total distance	338.25 ± 71.31	194.24 ± 101.78	0.0005	6	

References:
1. Spurney CF, Gordish-Dressman H, Guerron AD, et al. Preclinical drug trials in the mdx mouse: assessment of reliable and sensitive outcome measures. Muscle Nerve. 2009 May;39(5):591-602.
2. Rayavarapu S, Van der meulen JH, Gordish-Dressman H, et al. Characterization of Dysferlin deficient SJL/J Mice to Assess Preclinical Drug Efficacy: Fasudil Exacerbates Muscle Disease phenotype. PLoS One 2010; 5(9):e12981.
3. Sali A, Guerron AD, Gordish-Dressman H, et al. Glucocorticoid-treated mice are an inappropriate positive control for long-term preclinical studies in the mdx mouse. PLoS One. 2012;7(4):e34204.

Data exclusions

Lipid concentrations extracted from the LipidSearch software were further analyzed with an in-house script using the R programming language. The data was filtered to exclude any peak concentration estimates with a signal to noise ratio (SNR parameter) of less than 2.0 or a peak quality score (PQ parameter) of less than 0.8. If this exclusion resulted in the removal of two observation within a biological triplicate, the remaining observation was also excluded.

Replication

All experiments were repeated 3 or more times with similar results. All attempts at replication were successful.

Randomization

Subjects were randomly assigned to different groups

Blinding

For all the experiments the investigators were blinded to group allocation during data collection and analysis.

Reporting for specific materials, systems and methods

We require information from authors about some types of materials, experimental systems and methods used in many studies. Here, indicate whether each material, system or method listed is relevant to your study. If you are not sure if a list item applies to your research, read the appropriate section before selecting a response.

Materials & experimental systems

n/a	Involved in the study
☐	☒ Antibodies
☐	☒ Eukaryotic cell lines
☒	☐ Palaeontology and archaeology
☐	☒ Animals and other organisms
☒	☐ Human research participants
☒	☐ Clinical data
☒	☐ Dual use research of concern

Methods

n/a	Involved in the study
☒	☐ ChIP-seq
☒	☐ Flow cytometry
☒	☐ MRI-based neuroimaging

Antibodies

Antibodies used

Chkα (Chka) (1:1000, Abcam Cat#ab88053), Pparα (Ppara) (1:1000, Abcam, Cat#Ab24509), Pparb (1:1000, Biorad, Cat#AHP1272), Cpt1b (1:1000, Proteintech®, Cat#22170-1-AP), Chkβ (1:250, Santa Cruz, Cat#398957), GAPDH (1:1000, Cell signaling, Cat#2118), Pparg (1:500, Santa Cruz, Cat# sc-7273), anti-laminin antibody (Abcam, Cat# ab11575, 1:1000), goat anti-rabbit IRDye-800- or -680- secondary antibodies (1:20,000, LI-COR Biosciences, Cat#926-32211 and Cat#926-68071), anti-mouse m-IgGκ BP-CFL 790 (Santa Cruz, Cat# sc-516181).

Validation

Chkβ antibody, which is commercially available Chkβ (Santa Cruz, Cat#398957), was further validated in our lab by showing a band at desired molecular weight in the samples from Chkb+/+ mice and absence of the band in the samples from Chkb deficient mouse. Also, over expression of GFP-Chkb protein by lentiviral vectors at three different doses resulted in a band at expected molecular weight and expected intensities. The image is presented in the manuscript (Figure 2 and 5) and in the source data.

All the other antibodies have been widely used and cited in literature; Citations and images are also available from the companies website.
Chka antibody (ab88053) has been validated by the company to detect Chka protein in WB of mouse tissue and referenced in 9 publications: (PMID: 33574922, PMID: 32209482, PMID: 31788037, PMID: 31577958, PMID: 28566381, PMID: 27588131, PMID: 27705917, PMID: 27489281, PMID: 26490867).

Pparg antibody (Santa Cruz, Cat# sc-7273) is validated by the Santa Cruz company to react with mouse antigen for WB application and is referenced in 1030 publications; PMID: 35027935 PMID: 34984103 PMID: 34134993 PMID: 33617465, PMID: 34680177, PMID: 33479187 .
Gapdh antibody (Cell signaling, Cat#2118) is validated by the Cell signaling company to react with mouse antigen for WB application and is referenced in 4095 publications; PMID: 34738628, PMID: 34585157, PMID: 34760339, PMID: 33440995

Ppara antibody (ab24509) has been validated by Abcam company for western blot application and referenced in 69 publications to study Murine Ppara protein expression : PMID: 33769716 (rats), PMID: 33777674 (mice), PMID: 33919054(rats), PMID: 34029162 (mice), PMID: 33416097(rats) PMID: 33536915 (mice) PMID: 32404366 (mice) PPMID: 33299856(mice), PMID: 32966473(mice), PMID: 32292119 (mice).

Cpt1b antibody (Proteintech®, Cat#22170-1-AP) is validated by the Proteintech company to react with mouse antigen for WB application and is referenced in 22 publications; PMID: 30521848, PMID: 32110930, PMID: 34138236, PMID: 34606978, PMID: 34038646

Pparb antibody (Biorad, Cat#AHP1272)y, has been validated by Abcam company for western blot application and is expected to react with Mouse antigen. PMID: 15949697, PMID: 16511591,

Laminin antibody (Abcam, Cat# ab11575) is validated by the Abcam company to react with mouse antigen for IHC application and is referenced in 69 publications; PMID: 33145960, PMID: 33377198, PMID: 33507989, PMID: 33515576, PMID: 33643791, PMID: 33845891.

Goat anti-rabbit IRDye-800- or -680-secondary antibodies are validated by LI-COR company to be used for Western blotting application.

Mouse IgGκ light chain binding protein (m-IgGκ BP) conjugated to CruzFluor™ 790 (CFL 790) is a strongly recommended alternative to conventional goat/rabbit anti-mouse IgG secondary antibodies for Western Blotting (WB) and is referenced in 5 publications; PMID: 33908867, PMID: 33523588 , PMID: 30624730 , PMID: 29615473 PMID: 30422652 .

Eukaryotic cell lines

Policy information about cell lines

Cell line source(s)

We followed a protocol outlined in Shahini et al. 42 for isolation of myoblast by enabling the outgrowth of these cells from muscle tissue fragments of Chkb+/+ and Chkb-/- mice.

Authentication

The myogenic cell population was purified to ~97% with one round of pre-plating on collagen coated dishes, where differential attachment of fibroblasts and other non-myogenic progenitors separates them from myoblasts. The cells were positive for myogenic markers such as α7-integrin, MyoD, and Desmin.

Mycoplasma contamination	All cell line tested negative for mycoplasma contamination.
Commonly misidentified lines (See ICLAC register)	*Name any commonly misidentified cell lines used in the study and provide a rationale for their use.*

Animals and other organisms

Policy information about studies involving animals; ARRIVE guidelines recommended for reporting animal research

Laboratory animals	Chkb mutant mice in C57BL/6J background were a kind gift of Professor Gregory A. Cox and were originally generated at the Jackson Laboratory (Bar Harbor, Maine, USA) 5. Male Chkb+/- mice on the C57BL/6J background were crossed with female Chkb+/- on the same background to generate Chkb+/+, Chkb-/- and Chkb+/- littermates. The mutation identified in Chkb-/- mice is a 1.6 kb genomic deletion between exon 3 and intron 9 that results in expression of a truncated mRNA and the absence of Chkb protein expression.<br><br>For phenotyping experiments, 12 Chkb+/+, 12 Chkb+/- and 7 Chkb-/- mice from 6 weeks to 20 weeks of age were used . TEM analysis were performed on 12 and 115 days Chkb+/+ and Chkb-/- mice as described in the manuscript.<br><br>Lipidomics analysis were performed on muscle samples from 12 and 30 days old Chkb+/+ and Chkb-/- mice as described in the manuscript.<br><br>Western blot and RT qPCR analysis were performed on muscle samples from 30 days old Chkb+/+, Chkb+/- and Chkb-/- mice as described in the manuscript.
Wild animals	The study did not involve any wild animals.
Field-collected samples	The study did not involve samples collected from the field.
Ethics oversight	All animal procedures were approved by the Dalhousie University's Committee on Laboratory Animals in accordance with guidelines of the Canadian Council on Animal Care Guide to the Care and Use of Experimental Animals (CCAC, Ottawa, ON, Canada: vol. 1, 2nd ed., 1993; vol. 2, 1984.

Note that full information on the approval of the study protocol must also be provided in the manuscript.
